# Integrating evidence from research into decision-making for controlling endemic tropical diseases in South East Nigeria: perceptions of producers and users of evidence on barriers and solutions

**DOI:** 10.1186/s12961-019-0518-y

**Published:** 2020-01-13

**Authors:** Uchenna Ezenwaka, Chinyere Mbachu, Enyi Etiaba, Benjamin Uzochukwu, Obinna Onwujekwe

**Affiliations:** 1 Health Policy Research Group, Department of Pharmacology and Therapeutics, College of Medicine, University of Nigeria Enugu Campus, Enugu, Nigeria; 2 Department of Health Administration and Management, Faculty of Health Science and Technology, College of Medicine, University of Nigeria Enugu Campus, Enugu, Nigeria; 3 Department of Community Medicine, College of Medicine, University of Nigeria Enugu Campus, Enugu, Nigeria

**Keywords:** Evidence-based decision-making, Endemic tropical diseases, Evidence informed, HPSR, Use of evidence, GRIPP

## Abstract

**Background:**

Endemic tropical diseases (ETDs) constitute a significant health burden in resource-poor countries. Weak integration of research evidence into policy and practice poses a major challenge to the control of ETDs. This study was undertaken to explore barriers to the use of research evidence in decision-making for controlling ETDs. It also highlights potential strategies for addressing these barriers, including the gaps in research generation and utilisation in the context of endemic disease control.

**Methods:**

Information on barriers and solutions to integrating research evidence into decision-making for controlling ETDs in Anambra State, Nigeria, was collected from 68 participants (producers and users of evidence) during structured discussions in a workshop. Participants were purposively selected and allocated to groups based on their current involvement in endemic disease control and expertise. Discussions were facilitated with a topic guide and detailed notes were taken by an appointed recorder. Outputs from the discussions were synthesised and analysed manually.

**Results:**

Cross-cutting barriers include a weak research linkage between producers and users of evidence and weak capacity to undertake health policy and systems research (HPSR). Producers of evidence were purported to conceptualise and frame their research questions based on their academic interests and funders’ focus without recourse to the decision-makers. Conversely, poor demand for research evidence was reported among users of evidence. Another user barrier identified was moribund research units of the Department of Planning Research and Statistics within the State Ministry of Health. Potential solutions for addressing these barriers include creation of knowledge networks and partnerships between producers and users of evidence, institutionalisation of sustainable capacity-building of both parties in HPSR and revival of State research units.

**Conclusions:**

Evidence-informed decision-making for controlling ETDs is limited by constraints in the interactions of some factors between the users (supply side) and producers (demand side) of evidence. These constraints could be solved through stronger research collaborations, institutionalisation of HPSR, and frameworks for getting research into policy and practice.

## Background

Infectious diseases that occur solely or predominantly in hot, humid conditions are referred to as tropical diseases. Endemic tropical diseases (ETDs) encompass infectious diseases such as malaria, HIV and tuberculosis, whose endemicity/prevalence remains the same all year round. Neglected tropical diseases (NTDs) refer to a diverse group of tropical infectious diseases that receive relatively low funding and research attention, including lymphatic filariasis, onchocerciasis, leprosy, leishmaniasis, African trypanosomiasis and schistosomiasis [1]. In many resource-poor countries, ETDs still constitute a high annual health burden with resultant losses in economic productivity and social progress [2]. For instance, the World Malaria Report estimates that, in 2017, 219 million people were infected with *Plasmodium falciparum* malaria worldwide. Overall, 15 countries accounted for 80% of infections in sub-Saharan Africa (SSA) and India, and the highest numbers of infections were recorded in Nigeria (25%) and Democratic Republic of the Congo (11%) [3]. Similarly, the impact of NTDs on the health and economy of communities is gaining more international attention with a call for global efforts to eliminate or eradicate 10 NTDs by 2020 [4, 5]. In SSA, NTDs collectively produce a burden that might be equivalent to up to one-half of the malaria disease burden and more than twice that caused by tuberculosis in the region [6].

Evidence from research shows that, in 2013, 34 African countries were endemic to lymphatic filariasis, and Nigeria had the highest burden, with 80 to 120 million people at risk [7, 8]. In 2015, Nigeria contributed the highest burden (29 million) of schistosomiasis in SSA [9]. The country also has the greatest number of people infested with or at risk for hookworm (38 million), trichuris (34 million) and ascaris (55 million) in the sub-region [8–10]. In addition to these, Nigeria, like most sub-Saharan African nations, failed to meet Millennium Development Goal targets for malaria and other endemic diseases due to a multiplicity of health system-related, political and systemic challenges that contribute to increasing burden of endemic diseases and NTDs [11].

Poorly informed decision-making, particularly in low- and middle-income countries (LMICs), is one of the reasons why healthcare services at times fail to reach those most in need, why health indicators are poor and why many affected countries could not meet the Millennium Development Goals [12]. A major health system barrier to the effective control of ETDs is the lack of evidence-informed decision-making (EIDM), especially in health policy and systems research (HPSR), which is required to strengthen the health system and ensure optimal control of ETDs. This barrier is partly due to the scarcity of scientists and health professionals with relevant knowledge and expertise in communicable disease research and HPSR [13].

Contextualising evidence is a key challenge in effective policy-making and practice [14]. Ensuring that evidence from research is used for decision-making is essential to ensure that decision-makers develop and implement the right policies that will be effective and lead to significant improvement in service delivery outcomes [2]. Although evidence from research and its application in policy-making are promoted widely [15, 16], Nigeria recorded a failure to achieve the ‘research for health’ target of utilising research to inform policy and programmes by 2015, as stated in the first National Strategic Health Development Plan [17].

Many studies have shown that evidence from research can improve health systems effectiveness [18, 19]. Therefore, the significance of EIDM in LMICs amidst the diversity of healthcare needs should be promoted for effective, efficient and equitable strengthening of the health system [20, 21]. It is hence recognised that intervention/programmes are more effective if supported by research evidence; this also enables better value for money, transparency in decision-making and accountability. This means that incorporating evidence from research into decision-making is critical for health systems responsiveness and successful implementation of endemic disease control programmes [22].

There are gaps in understanding the constraints in using evidence from research for decision-making in endemic disease control in Nigeria. These gaps could be attributed to context-specific organisational and system-wide barriers that are rooted in both the generation of research evidence and integrating research evidence in decision-making. Unless these barriers are addressed, strategies to boost the capacity of policy-makers and programme managers to access and use research will have limited outcomes [23]. Pang et al. [24] propose a framework of health research systems that aim for advancement and utilisation of scientific knowledge for health systems improvement; this framework describes four principal functions, namely stewardship, financing, creating and sustaining resources, and producing and using research.

This study was undertaken to explore barriers to the use of research evidence in decision-making for the control of ETDs. It also identifies potential strategies for addressing gaps in research generation and utilisation for endemic disease control. This paper contributes to knowledge of organisational and systemic barriers to EIDM. It also provides information on potential solutions for addressing these barriers to facilitate getting research evidence into policy and practice (GRIPP) for control of ETDs. This is particularly important because, as global funding for malaria and other ETDs increases, EIDM is required to ensure the achievement of cost-effective and efficient reductions in disease burden [4, 25].

## Methods

### Study design and study setting

This was a cross-sectional study conducted in Anambra State, Nigeria, which explored stakeholders’ perspectives of barriers to the use of evidence in decision-making for the control of ETDs in the State. The State is located in the south-eastern part of Nigeria and had a projected population of 5,684,655 by the end of 2017. In Anambra State, the State Ministry of Health (SMoH) coordinates all health activities in the State.

### Study population

The study population consisted of producers and users of evidence drawn from tertiary institutions and health organisations in Anambra State.

Producers of evidence (also known as researchers) are involved in HPSR and other research evidence generation. Some of them also teach in tertiary institutions in the State. They were drawn from the Departments of Community Medicine, Nursing Science, Pharmacy, Medical Laboratory Sciences, and Parasitology and Entomology from tertiary institutions in the State, namely Nnamdi Azikiwe University, with three campuses located at Awka, Nnewi and Agulu, and Chukwuemeka Odumegwu Ojukwu University, with three campuses located at Uli, Igbariam and Awka.

Users of evidence were policy-makers, senior healthcare managers and programme officers who use evidence from research in decision-making and health programming. They were selected from various departments/units in the SMoH and health agencies such as the Department of Public Health/Disease Control, the Department of Planning Research and Statistics (PRS), the Department of Pharmacy, and the Health Administration and Management unit.

The participants were purposively selected to ensure diversity in organisation and expertise. The specific participants included directors of public health, medical services, pharmacy and PRS, heads of the Local Government Department of Health, the executive secretary of State Primary Health Care Development Agency, the hospital administrator of the State Hospital Management Board, a State epidemiologist, project managers of ETDs (such as Malaria and other NTDs), and data management officers. Other users of evidence selected from outside the health sector were from the State Ministry of Economic Planning and Budgeting because they play a vital role in planning, budgeting and budget approval. Table 1 highlights the socio-demographic and other characteristics of the participants.

**Table 1:** Background characteristics of producers and users of evidence in Anambra State, Nigeria

Producers of evidence (*N* = 32)	
Gender	
Male	15
Female	17
Organisation	
NAUTH Nnewi	26
NAU Awka	4
COOUTH Awka	2
Department/Unit	
Community medicine	19
Laboratory science/services	3
Pharmacy	5
Nursing/Midwifery	5
Academic/Professional cadre	
Registrar	15
Consultant/Lecturer	12
Professor/Reader	3
Others (Postgraduate student)	2
Users of evidence (*N* = 36)	Freq.
Gender	
Male	20
Female	26
Organisation	
SMoH	23
SPHCDA	7
Others^a^	6
Department/Unit	
Endemic disease control	14
PRS	5
Medical/health services	7
Role/Designation	
Departmental/Unit head	6
Directors/Executive Secretary	5
Programme Manager	4
M&E Officer/Programme analyst/Statistician	6
Higher Executive Officer	5
Planning Officer/Accountant/Logistician	8
Nursing officer	2

^a^Others – Anambra state Ministry of Economic Planning and Budgeting, School of Nursing services

*COOUTH* Chukwuemeka Odumegwu Ojukwu University Teaching Hospital, Awka Anambra State, *NAU* Nnamdi Azikiwe University Awka, *NAUTH* Nnamdi Azikiwe University Teaching Hospital Nnewi, Anambra State, *M&E* monitoring & evaluation, *PRS* Planning Research and Statistics, *SMoH* State Ministry of Health, *SPHCDA* State Primary Health Care Development Agency

### Data collection

Data were collected during group discussions in two separate workshops. The first workshop was organised for experts in endemic disease control in Anambra state. Twenty-one participants (11 producers and 10 users of evidence) attended the workshop and comprised senior healthcare managers, endemic disease control programme managers, heads of research units in tertiary institutions, and academicians who had contributed significant research evidence for endemic disease control. The second workshop was organised for early career researchers, mid-level managers and ETD control programme officers in the State. There were 47 participants – 22 producers and 25 users of research evidence. The workshop was designed in the form of parallel sessions of 2-day meetings with the two categories of participants. The first workshop was held in October 2016 while the second one was held in February 2017. Each workshop lasted 2 days. Structured group discussions were used to explore participants’ perceptions and experiences (as individuals and a collective) of EIDM for the control of ETDs. Each group had 5/6 participants. Discussions were moderated by research team members with expertise in qualitative research, HPSR and endemic disease research. Each group discussed the following questions: In what way does your organisational structure support evidence-based decision-making for endemic disease control? What individual, organisational and systemic factors enable or constrain translating research evidence into policy and practice in your organisation and in what ways? How could the barriers be mitigated for better endemic disease control? What structures should be developed in the SMoH to entrench research and GRIPP in the State?

Group discussion was followed by feedback to the plenary of highlights or key findings. Detailed notes of group discussions and comments from the plenary discussion were synthesised into transcripts for analysis.

### Data analysis

Thematic content analysis was performed manually. Data were categorised based on recurrent or common themes to present key elements of participants’ accounts [26]. In-depth reading of textual data, to gain an overall understanding of participants’ views was followed by identification and linking of ideas to generate themes for coding. Major themes and sub-themes used in coding responses were (1) systemic barriers to EIDM, namely research linkage, capacity to undertake and use research evidence, functionality of research units and research ethics committees, poor government funding of research, weak health management information systems (HMIS), and political interference; (2) organisational barriers to EIDM, namely decision space of users of evidence and poor demand and support for research evidence; (3) potential strategies/solutions for mitigating barriers to EIDM, namely creation of networks for stronger linkage, continuous and sustainable capacity-building, revival of research unit in SMoH, institutionalisation of a State health research and ethics committee (SHREC), fund generation for research, recreation of information communication and technology (ICT) research centre; and (4) enablers to potential solutions, namely established linkage between users and producers of evidence, built capacity on EIDM, high political will and commitment, implementing support, etc.

## Results

### Systemic barriers to integrating research evidence into decision-making processes

Systemic barriers to EIDM for endemic disease control in the state, identified by respondents, are presented in Table 2 below.

**Table 2:** Summary of perceived barriers to EIDM and potential solutions

Barriers to EIDM	Potential solutions to identified barriers
Weak linkage and networking between researches and users of evidence	Creation of networks that will ensure a strong linkage between producers and users of evidence
	• Involvement of both producers and users of evidence in research conceptualisation, evidence generation and dissemination
	• Users of evidence communicating identified problems to producers of evidence
	• Creation of knowledge exchange forum among users and producers of evidence
	• Institutionalising exchange programmes and feedback strategy through meetings and workshops • Collaborating with existing research institutions such as the Health Policy Research Group
Poor demand and support for research evidence	Creation of a supportive research evidence environment
	• Advocacy for demand and supply-driven research
	• Collaboration between research institutions and users of evidence
	• Increased uptake of research findings by users of evidence (to motivate producers)
	Promulgate legislative back up for integrating research evidence in decision-making
	• Developing and implementing an evidence-based framework in SMoH
Weak capacity to undertake and use research evidence	Continuous and sustainable capacity-building on EIDM
	• Sustainable capacity-building workshops on EIDM
	• Continuous training on data management for officers for M&E, HMIS and planning officers
Moribund research unit in Department for PRS	Revival of the research unit in Department of PRS
	• Recruitment of health systems researchers and health economists in the department to enable translational research
	• Conducting research and evaluation of implemented programmes
	• Periodic/annual research review meetings
Non-existence of SHREC	Institutionalisation of SHREC
	• Ensure and coordinate ethical conduct in health research in the state among other functions
	• Constitution of a technical working group for research
Lack of funds	Fund generation
	• State budgetary allocation for health research and programme evaluation
	• Advocating/sourcing funds for health research from donor agencies, philanthropists, etc.
Weak HMIS	Creation of ICT research centre for strengthening HMIS
	• Creating a functional database for storing health data and research evidence
	• Establishing a central evidence repository website
	• Recruitment and training of medical record officers in health facilities
	• Harmonising facility data collection tools across health facilities
Political interference	Minimise political interference
	• Media sensitisation on a need to reduce the occurrence and consequences of political inference and nepotism in the health sector
Limited decision space of users of evidence	Adopting/developing an evidence-based framework in SMoH

*EIDM* evidence-informed decision-making, *HMIS* health management information system, *ICT* information communication and technology, *M&E* monitoring and evaluation, *PRS* Planning Research and Statistics, *SHREC* State Health Research Ethics Committee, *SMoH* State Ministry of Health

#### Weak research linkages between producers and users of evidence

Weak linkage between research organisations and policy-making bodies was reported as a key barrier to knowledge translation and uptake of research evidence for decision-making. This was attributed to some cross-cutting and actor-specific factors. Cross-cutting factors, such as poor communication between producers and users of evidence, and inability to form and sustain research networks resonated among both categories of respondents. Users of evidence stated that there is a “*huge communication gap between users and producers of evidence*” and that research collaboration for endemic disease control is low as a result of “*poor networking among users and producers of evidence*”.

Actor-specific reasons (attributed to users of evidence) for weak research linkage include lack of interest and appreciation for the value of research evidence in decision-making. Producers of evidence were of the view that “*Policy and decision-makers do not appreciate or attach any relevance to health research evidence*”*.* They were also of the opinion that users of evidence exhibit extreme territorialism when it comes to joint decision-making, often dismissing researchers and stating that they should focus on academic advancement while they (policy-makers) manage the health sector.

Actor-specific reasons attributed to producers of evidence for weak linkages include irrelevant research priorities and inaccessible research outputs. Users of evidence perceived their counterparts to conduct research that suits their interests and, often, these do not match research priorities for endemic disease control. This is probably due to the “*non-involvement of users/appropriate decision-makers in research priority setting*”*.* Furthermore, evidence generated from research is often communicated through journal publications for academic/professional development, and policy-makers do not find these journals accessible nor the language of communication understandable.

#### Poor capacity to undertake and use research evidence

Weak research capacity and opportunities for mentorship were identified and emphasised by both users and producers of evidence as barriers to EIDM. Users of evidence identified the lack of deep understanding of research, the processes involved and its application in decision-making as a challenge. Similarly, producers of evidence affirmed that poor capacity to undertake health systems research as well as a lack of opportunities for mentorship deter integration of evidence in planning and implementation of health programmes, as stated: “*Poor knowledge of HPSR, GRIPP, and lack of mentorship*”*.*

#### Functionality of research units and research ethics committees

Another significant finding was the moribund state of the research unit of the Department of PRS in the SMoH, whose responsibilities include planning of health programmes, collating and analysing health data, conducting research, evaluating health programmes or interventions, and dissemination of data for planning of health programmes. The department does not conduct or coordinate research projects. As stated: “*Moribund Research unit; the ‘R’ in PRS is silent; they do not conduct research*”, these barriers were perceived to have contributed to the existing communication gaps and poor collaboration amongst producers and users of evidence. The department also lacks adequately qualified human resources, leading to constrained data management activities – “*PRS department is understaffed*”*.*

At the time of the study also, there was no SHREC to coordinate and provide ethical clearance for the conduct of research.

#### Poor government funding to research

Lack of funding for research emerged as another barrier. Budgetary allocation to research activities (training, capacity-building, etc.) is not prioritised in the state, and even when it is included in the budget, the release is poor and hindered by bureaucracy – “*No budgetary allocation/funds for research activities*”*.* More so, they noted that “*bureaucracy in the budget release is envisaged to be another big issue*” when such funds are allocated in the state budget. Producers of evidence also experienced a lack of institutional support for research. Most of their support came from external grants and self-sponsored research projects. Most “*research done is funded by international agencies*”*.* Producers felt that if users attached more importance to research evidence, it could lead to better allocation and release of funds for research from the State budget.

#### Weak Health Management Information System

Data generated monthly from primary and secondary health facilities on service utilisation serve as the main source of health data in the State. However, participants reported that several data records are incomplete or unavailable, and there are discrepancies across data sources. There were reports that the multiplicity of reporting tools/registers for various programmes and development partners makes a harmonisation and comparison of data from different sources and tools difficult for analysis and utilisation in decision-making. Some participants also highlighted a lack of functional database and the absence of ICT centres, which hinder data management and storage.

#### Political interference and nepotism

Political interest determines the ‘method’ and ‘content’ of a set of decisions made, a programme to be implemented and thematic area for implementing the preferred programme. Although users of evidence are charged with the day-to-day planning and implementation of endemic disease control programmes, politicians ultimately determine ‘how’ and ‘what’ policies or decisions to take up. “*Political interference in the health sector affects how decisions are made in health the sector*”*.*

Participants perceived nepotism as a challenge to the use of research in decision-making and planning of programmes. Employment and appointments to strategic positions in the health sector are made by politicians. Such positions are given to friends, relatives, special-interest groups or used as political settlement particularly to people who campaigned for them during an election. These groups of persons sometimes are not competent to manage such positions. Therefore, they are most times “*unwilling and are resistant to change due to personal interest/gains*” and “*apathy*”. Consequently, they usually adopt a negative attitude towards moving from the anecdotal evidence-based method of planning health programmes to a systematic EIDM.

### Organisational barriers to EIDM

Participants identified some organisational level barriers to EIDM for endemic disease control.

#### Limited decision space of users of evidence

There is no organisational strategy or guideline or system for integrating research evidence into policy-making in SMoH, and potential users of evidence have limited decision space to change this. The existing ‘top-bottom’ decision-making approach excludes relevant actors at all levels of care and, consequently, leads to poor implementation outcomes. “*Our system is not structured in such a way that evidence will be used for making decisions*”.

#### Poor demand and support for research evidence

Producers of evidence were of the opinion that, because users of evidence do not attach much importance to research evidence, their demand for evidence was sub-optimal. “*Policy-makers do not appreciate the importance of research evidence, which is why there is poor demand*”. Similarly, users of evidence acknowledged little or no demand for research evidence when planning and implementing ETD programmes in the State. “*There is a thick wall between researchers and users of evidence in terms of communicating and demanding for research evidence*”.

#### Potential solutions for mitigating identified barriers to EIDM

Participants highlighted some strategies that could potentially reduce barriers to EIDM for control of ETDs in Anambra State. These strategies are presented below.

#### Creation of networks for ensuring a strong linkage between researchers and users of evidence

Participants suggested that poor interaction between users and producers of evidence could be enhanced through better engagement in the research process, advocacy for demand-driven research, and establishment of knowledge exchange channels to improve communication and information sharing between producers and users of evidence. Some quotes that highlight participants’ suggestions of how to improve linkage between researchers and users of evidence include “*Involvement of both producers and users of evidence from research conceptualisation to research evidence production*”, “*advocacy for demand and supply-driven research*”, “*creation of a knowledge exchange forum (formal or informal) between users and producers of evidence*”, *and* “*feedback strategy, that is, dissemination of research evidence through meetings and workshops*”.

#### Sustainable capacity-building on the use of evidence in decision-making

Capacity-building interventions through workshops and continuous training were suggested by some participants for improving EIDM. “*There is need for continuous training with a focus on improving the capacity of decision-makers to understand and use research evidence*”.

Specific capacity-building workshops on EIDM, HPSR and GRIPP were mentioned by a participant. Another person highlighted the need for continuous training of data management officers in monitoring and evaluation, HMIS and PRS units of the SMoH.

#### Revival of the research unit in the Department of PRS

Participants suggested that, in order to revive and strengthen the moribund research unit of Department of PRS in the SMoH, the government needs to recruit health systems researchers and health economists to enable the evaluation of health interventions/programmes and implementation of translational research and HPSR.

#### Institutionalisation of SHREC

Participants’ suggested the “*establishment of SHREC to ensure and coordinate the ethical conduct of health research*”*.* Another person suggested the constitution of a technical working group (TWG) for research to coordinate research activities going on in the State. This TWG would comprise researchers from tertiary institutions and users of evidence from the SMoH and relevant health agencies.

#### Fund generation for health research

To avert the challenge of poor research funding, participants recommended “*budgetary allocation for health research and programme evaluations*” as well as “*advocating/sourcing funds for research from different sources such as donor agencies, local NGOs, philanthropists etc.*”.

#### Promulgate legislative back up for integrating research evidence in decision-making

Additional to creating a conducive environment for research, participants suggested that the government should establish a law that mandates EIDM in health. This would also entail establishing a structure within the SMoH that ensures research evidence is embedded in decision-making processes for better control of ETDs. “*Creating an environment and system for research use, notably developing and implementing a legislative document or framework for optimising evidence use in decision-making*”*.*

#### Creation of ICT research centre

Participants highlighted the need to create an ICT centre with a functional database to strengthen HMIS. “*ICT centre and database and media platform for storage and dissemination of research findings*”, “*a centralised repository of evidence for knowledge sharing and a State website for research evidence conducted in the State*”, and “*harmonising facility data collection tools across health facilities for easy and better data analysis and comparison*” were stated as required.

#### Minimising political interference

Participants suggested that, in order to minimise political interference in the health sector, there needs to be “*sustained media campaign on consequences of political interference and nepotism and how this can be reduced in the health sector*”.

### Current enablers to potential solutions

Several factors were identified by participants to facilitate the effective use of research evidence in decision-making. Participants expressed optimism about the feasibility of these potential solutions leveraging on the following enabling factors: (1) existing relationships and linkage with the Health Policy Research Group; (2) improved individual competence and organisational capacity for EIDM; (3) good political will and commitment to health sector improvements by the current State governor; (4) increasing interest to integrate research evidence in decision-making processes among users and producers of evidence; (5) active engagement of decision-makers in the Ministry of Economic Planning and Budgeting to facilitate the release of budgetary allocations to health; (6) availability of donors/partners in NTDs and malaria control in the State, (e.g. The Carter Center in collaboration with Research Triangle Institute, GOWON Foundation, WHO, UNICEF); and (7) willingness to create a knowledge translation forum between users and producers of evidence.

## Discussion

This study reveals several barriers to the utilisation of research evidence in decision-making for efficient and effective control of malaria, NTDs and other ETDs in Anambra State. It also highlights participants’ perceptions of potential solutions for improving EIDM in control of ETDs in the State. The barriers to utilisation of research evidence which are highlighted in this study corroborate findings from other studies that have examined evidence-based decision-making in health [27–31].

The weak linkage that was reported to exist among producers and users of research evidence underscores a communication gap in research priority-setting. It has been stated that, more often than not, research priorities of researchers align with the personal interest of funders, rather than the needs of decision-makers and policy-makers [27]. The level of engagement and interaction between producers and users of evidence could influence knowledge exchange and sharing, which are critical for bringing about change in policy and practice. Previous studies have reported that continuous and sustained engagement, collaboration and participation by researchers and policy-makers enables the acceptability of research evidence and enhances translation of research into policy and action [2, 28]. Fostering collaboration between producers and users of evidence has been reported as a key factor in bridging evidence to policy gaps [32]. It is therefore beneficial to continuously involve users of evidence in research priority-setting, implementation and dissemination.

The poor demand for research evidence among users of evidence was also reported as a barrier to EIDM for control of ETDs, as it has been in other studies undertaken in LMICs, and it may reflect policy-makers’ perception of the value of research evidence in endemic disease control or their prioritisation of research for decision-making as a whole. Other reasons reported in the literature for the poor demand of research evidence by policy-makers in LMICs include weak collaboration with producers of evidence, poor understanding of research evidence and its application, and weak technical capacity to integrate research evidence into policy and practice [29, 33–35]. Research capacity at different institutional levels and interfaces of evidence generation and use in policy- or decision-making has been identified as the major strategic issue in HPSR because weak capacity to use evidence from research constrains integration of research evidence into decision-making [32]. The way research evidence is communicated could also influence demand by policy-makers. It has been argued that some users of evidence might not have the proficiency and resources to access research evidence or the time to source for this evidence from scientific journals [30]. Presenting research findings in less complex formats, such as policy briefs that adopt simple language, has been shown to improve research uptake by policy-makers [31, 33].

The absence of structural enablers of EIDM were perceived by many participants as a major hindrance to utilisation of research evidence for control of ETDs in Anambra State. The lack of budgetary allocation for research in Anambra State health budget conforms to findings from developing countries, including Nigeria, which have reported inadequate funding as a challenge to HPSR+A [36, 37]. Although state-funded research is more likely to generate valuable and relevant findings for policy and practice, its capital-intensive and complex nature may hinder investment in research by policy-makers. The absence of supportive structures for getting research evidence into policy and practice in the health sector also manifested in the non-existence of a State Health Research Ethics Committee coupled with a non-functional research unit in the Department for PRS. This has implications for resource allocation in planning and implementation of ETD programmes because inefficient utilisation of limited resources for disease control could result in poor health outcomes and failure to meet health targets/goals. Establishing SHREC and reactivating the research unit could contribute to entrenching research into decision-making for better control of endemic diseases in Anambra State. It has been noted that local ethical review boards are required to offer research oversight and ensure that research studies are relevant and conform to International Ethical Standards [38].

The participants shared their experiences of political interferences during planning and implementation of health programmes for control of endemic diseases. This politicised nature of decision-making was considered a barrier to integrating research evidence into decision-making for control of ETDs. A report shows that at any organisational level, political interferences and powers continually hinder the delivery of HPSR evidence and its application in decision-making [39]. This necessitates the need to institute effective approaches for reducing adverse political interference in decision-making.

Poor quality of data generated from health facilities is another challenge to the use of research evidence in decision-making. Existing data in health facilities were reported to be incomplete and inconsistent, which make data analysis and usability (for programme evaluation and decision-making) almost impossible [40]. Poor data quality from primary health facilities has been attributed to inadequate human resources for health and weak capacity to analyse and manage health data at the state and local government levels [40]. In the absence of trustworthy and usable data, programme planning for ETDs will be done abstractly without adequate consideration of context- and population-specific concerns and challenges.

In concordance with some of the solutions that were proposed in our study, a previous study in a similar setting described four strategies for getting research evidence into policy and practice, namely increased demand for research evidence by policy-makers, involvement of users of evidence in research priority setting, design and implementation, facilitating researcher–policy-maker engagement through workshops and research networks, and active dissemination of research evidence to relevant policy-makers and other stakeholders [28]. Therefore, promoting ownership of research findings and strengthening knowledge exchange and communication through policy-maker–researcher linkages are imperative for getting research evidence into policy and practice for the control of ETDs [42, 43]. It is also recognised that developing and investing in a national health research capacity is a key element for strengthening health systems [16].

In conclusion, the current decision-making process for control of ETDs is not significantly influenced by research evidence due to various organisational and system barriers such as weak linkage between users and producers of evidence, poor demand for research evidence, weak capacity to generate and/or use research evidence, poor data quality and absence of structural enablers such as funding and frameworks for EIDM. Potential solutions for addressing these barriers include the creation of knowledge networks and partnerships between producers and users of evidence, institutionalisation of sustainable capacity-building of both parties in HPSR and revival of state research units. Embracing these solutions could significantly improve the planning and implementation of ETD control programmes as well as health systems performance.

## Data Availability

The dataset used for this study is available and can be obtained from the corresponding author upon request.

## Abbreviations

EIDM: evidence-informed decision-making
ETDs: endemic tropical diseases
GRIPP: getting research evidence into policy and practice
HMIS: health management information system
HPSR: health policy and systems research
ICT: information communication and technology
LMICs: low- and middle-income countries
NTDs: neglected tropical diseases
PRS: planning research and statistics
SHREC: State Health Research Ethics Committee
SMoH: State Ministry of Health
SSA: sub-Saharan Africa
